# Social support and HIV/STDs infections among a probability-based sample of rural married migrant women in Shandong Province, China

**DOI:** 10.1186/s12889-015-2508-5

**Published:** 2015-11-24

**Authors:** Wenkang Ma, Dianmin Kang, Yapei Song, Chongyi Wei, Gifty Marley, Wei Ma

**Affiliations:** Department of Epidemiology and Health Statistics, School of Public Health, Shandong University, 44 West Wenhua Road, Jinan, Shandong Province 250012 China; Institute of AIDS Control and Prevention, Shandong Center for Disease Control and Prevention, Jinan, China; Nosocomial Infection Control Section, Zhengzhou No.7 People’s Hospital, Zhengzhou, China; Department of Epidemiology and Biostatistics, The University of Californian, San Francisco School of Medicine, San Francisco, USA

**Keywords:** HIV/AIDS, Migrant women, Social support, China

## Abstract

**Background:**

The increasing population of marriage-based migrant women is disproportionally affected by AIDS/STDs in China, and social support plays a critical role. This study aims to describe the social support level received by married migrant women in rural areas in Shandong province in comparison to non-migrant local women, identifies the relevant factors of this social support condition among married migrant women, and observes the correlation between social support level and infection status of AIDS and STDs among this group.

**Methods:**

A probability-based sample of 1,076 migrant and 1,195 local women were included in the study. A pre-tested field questionnaire was administered to participants through a direct face-to-face interview. Questionnaire contained questions on socio-demographic information, AIDS and STDs prevalence information and Social Support Rating Scale (SSRS) which measures objective support, subjective support, and utilization of social support.

**Results:**

Compared to local women, married migrant women had lower levels of social support in most dimensions. Multi-variable analysis revealed that relationship with spouse, family average income, number of children, education, engagement and claimed reasons of moving have various correlations with one or all dimensions of social support scores. Higher social support is also related to awareness of infection status of HIV and STDs among this group.

**Conclusion:**

Our findings provide further evidence that married migrant women have lower levels of social support which may be related to some social characteristics and their awareness status of AIDS and STDs infection status and that targeted interventions need to be developed for this population.

## Background

Migrants are broadly defined as people who move from one place to another temporarily, seasonally or permanently for a range of voluntary and/or involuntary reasons [1]. They play a critical role in the dynamics of infectious diseases like HIV [2–4]. Migration can have negative impact on mental health, making immigrants more vulnerable to mental health problem such as depression [5], which may increase risk behaviors associated with diseases such as HIV/AIDS. Moreover, migrants often have difficulty maintaining their health because of limited access to local healthcare as well as defective official supervision in host area [6, 7]. As a result, migrant populations can be disproportionately affected by certain disease outbreaks.

In 2011, it was estimated that approximately 780,000 people were living with HIV/AIDS in China. One of the characteristics of the HIV epidemic in China is that some sub-populations, such as migrant women, are disproportionately affected [8]. In 2009, 20 % of the 2,660 HIV/AIDS cases reported in Shandong province were among migrant women [9]. Due to a high male-to-female sex ratio and a growing gap in poverty, poor rural men who cannot afford to marry local women in their provinces seek to marry women from even poorer regions. These women usually come from poverty-stricken rural ethnic areas in Southwest China (such as Yunnan province, Guizhou province, and Sichuan province) and bordering countries (such as Burma and Vietnam) where HIV prevalence is higher and concentrated among key populations such as people who inject drugs [10, 11]. Epidemiological surveys found that HIV prevalence among these married rural migrant women in Shandong province was 0.65 % in 2008 [11], which is much higher than that among the general population in the province (range 0.01–0.09 %) [12, 13]. Among those who were HIV-positive, 20.4 % had an HIV-infected spouse and the rate of mother-to-child transmission was high (13.7 %) [11]. This high rate of mother-to-child suggested that HIV testing was not routinely conducted with pregnant migrant women; and hence a significant proportion of them may not be aware of their HIV status, which place their spouses and sexual partners at greater risk for infection [9].

It is hypothesized that factors such as demographic characteristics, an individual’s cultural competence [14], perceived discrimination [15] and acculturative stress [16, 17] interact with each other to affect psychological health among migrants. Among the many aspects of psychological health, social support plays a critical role and is an important indicator of migrants’ psychological well-being. Social support as a broad term normally refers to the perception and actuality that one is being cared for and has assistance available to him or her from other people [18]. It has been found to have positive effects on migrants’ health status as well as to enhance their utilization of health care services. For example, social therapeutic network, which utilized social network for health promotion, has been reported to benefit migrant women in pregnancy [19], and inadequate social support has been noted as a risk factor for poorer utilization of maternity services [20].

As such, availability of adequate social support or the lack of it may affect HIV risks among married migrant women in rural China. Despite the fact that migrant women bear a higher burden of HIV/AIDS compared to the general population in China, few studies have documented the social support profiles of this group. One descriptive study using cluster sampling reported lower levels of social support among these women compared to that among their local counterpart in a county in Shandong province [21]. As the number of women migrating to rural areas for marriage purposes is likely to increase [9, 10, 22], it is therefore important to examine levels of social support and associated risk factors among this population on wider scales and from representative samples so that appropriate intervention measures can be implemented to reduce HIV infections among these women and their spouses and sexual partners, as well as to improve their psychological well-being. Using a probability-based sample of married women from Shandong province, this paper describes levels of social support among rural migrant women in comparison to non-migrant local women, and identifies factors influencing social support among this group. It also investigates the correlation between levels of different social support dimensions and awareness of AIDS and STDs infection.

## Methods

### Participants recruitment

To increase diversity of participants (i.e., geographic distribution), three cities in Shandong Province, Yantai, Weifang, and Linyi, were selected to recruit participants. Yantai, Weifang and Linyi are located in the eastern, central and southern parts of the province, respectively. Two counties in each city and two towns in each of these counties were then randomly selected as study sites. Migrant and local non-migrant women were recruited using cluster sampling with migrant women being mostly scattered in different villages. Because there is no household registration or record of migrant women, nor are there any distribution patterns of these women, we recruited participants using the official checkup system. The township governments in Shandong Province provide free health checkups for women of child-bearing age at least once a year, hence for sampling efficiency participants were recruited from the population of women who attended the medical checkups. For the purpose of our study, we made several restrictions in our participant selection. First, we selected from women from areas with high HIV prevalence in southwest China (Yunnan province, Guizhou province, and Sichuan province) and bordering countries (Burma and Vietnam), because these places are the main sources of rural migrant women in Shandong [10, 11], and that in this way we can better assess the possible relationship between social support and HIV/STDs infection status. According to a report in 2011, 90 % of the HIV/AIDS patients in rural Shandong area had the age of 20–39 years old [9]. Thus, we chose participants from women who migrated to Shandong after the year 1990.

A total of 1,300 married migrant women qualified to partake in the study out of which 1,164 of them completed the interview (response rate of 89.5 %). Inclusion criteria for migrant women included: (1) was originally from a province or a foreign country with higher HIV prevalence such as Yunnan province, Guangxi province, Guizhou province, Sichuan province [8], Burma and Vietnam; (2) migrated to Shandong province after 1990, and was married to or cohabiting with a rural man in Shandong province, and (3) aged between 18–50. Inclusion criteria for non-migrant women included: (1) originally from Shandong province, (2) married, and (3) aged between 18–50. The Ethics Review Committee (ERC) of Public Health at the Shandong University has reviewed and approved the proposed use of human subjects in this research project.

### Data collection

After obtaining verbal informed consent from participants, face-to-face interviewer-administered survey interviews were conducted by trained female interviewers between September 2012 and January 2013. Socio-demographic characteristics, HIV related behaviors and social support information were collected. Socio-demographic information included current address, age, educational level, ethnicity, marriage history (first-married vs. remarried), annual income per capita in the household, quality of relationship with spouse, living arrangement (not living with family vs. living with family), total number of children during lifetime and that with current spouse, occupation, and time since migrating to Shandong Province. HIV-related behaviors included history of blood transfusion, premarital sex, extramarital sex, sexually transmitted diseases, drug use and HIV testing. Information on HIV and STDs infection was collected through questions as: Are you infected with HIV? a. Yes, b. No, c. I don’t know. Have you been diagnosed STD? a. Yes, b. No.

Social support was measured using the Social Support Rate Scale (SSRS), which has demonstrated good reliability among Chinese populations [23, 24]. SSRS is a 10-item scale measuring 3 dimensions of social support: subjective support (4 items), objective support (3 items), and utilization of social support (3 items). Questions on subjective support include questions such as: How many friends do you have who can provide help and support to you? a. none, b. 1–2, c. 3–5, d. 6 and more. Questions on objective support include: What have been your sources of economic and practical support in case of emergency? (1) no sources; (2) the following sources (multiple choice): a. spouse, b. other family members, c. relatives, e. colleagues, f. employer/company, g. official or semi-official bodies (e.g. political parties, or a labor union) h. unofficial bodies (e.g. religious groups, social groups or other social organizations), i. others (please identify). Questions on utilization of social support include: How do you seek for help when you are in trouble? (1) depending on my own and turning to no one, (2) seldom turning to others for help, (3) sometimes seeking help from others, (4) turning to families, relatives and some organizations. Scores are calculated and appraised on three dimensions as well as on a whole. The possible total score of SSRS ranges from 11 to 66 points. A higher score indicates greater social support. Cronbach’s alpha is 0.732 for the entire sample, and 0.707 and 0.741 for migrant women and local women, respectively.

### Data analysis

Responses with missing data were identified as invalid and excluded; and the analysis contained valid responses from 1,076 married migrant women and 1,195 local non-migrant women (the proportion of missing data was ≤25 %). Data were double entered with Epidata 3.1 and analyzed using SAS 9.1. Chi-square tests were conducted to compare socio-demographic characteristics between married migrant women and non-migrant women. Levels of social support between married migrant women and non-migrant women were compared using student *t* test. One-way analysis of variance (One-Way ANOVA) and multi-way analysis of variance (multi-way ANOVA) were conducted to identify factors significantly associated with receipt of social support, and dimensions of social support related to infection of AIDS and STDs among migrant women.

## Results

### Characteristics of participants

There were no significant differences between non-migrant women and migrant women by location of residency, age, perceived relationship quality with spouse and total number of children during lifetime (Table 1). However, migrant women were more likely to be illiterate to be ethnic minorities, and to report lower annual income per capita in the household compared to non-migrant women (*Ps* < 0.001). Migrant women had significantly fewer children with their current husbands, not living together with their husbands’ families, and were more likely to report that their current marriage was not their first marriage compared to non-migrant women (*Ps* < 0.001).

**Table 1:** Comparison of characteristics between non-migrant women (*N* = 1,195) and migrant women (*N* = 1,076) from rural area of Shandong Province, China

	Local women (*n* = 1,195)	Migrant women (*n* = 1,076)	*χ* ^2^	*P*
	*n* ^a^	*n* ^a^		
Current address				
Yantai	379 (31.7 %)	350 (32.5 %)	3.304	0.192
Linyi	397 (33.2 %)	386 (35.9 %)		
Weifang	419 (35.1 %)	340 (31.6 %)		
Age (year)				
<30	251 (21.6 %)	245 (23.0 %)	0.671	0.715
30–39	556 (47.8 %)	499 (46.9 %)		
≥40	355 (30.6 %)	319 (30.2 %)		
Education level				
Illiterate	23 (2.0 %)	198 (19.0 %)	434.648	<0.001
Elementary school	238 (20.5 %)	464 (44.5 %)		
Junior middle school	713 (61.5 %)	343 (32.9 %)		
Senior meddle school or above	185 (16.0 %)	37 (3.6 %)		
Ethinicity				
Han Chinese	1130 (99.2 %)	533 (52.0 %)	675.834	<0.001
Ethnic minorities	9 (0.8 %)	492 (48.0 %)		
Marriage history				
First marriage	1085 (97.3 %)	833 (84.3 %)	110.302	<0.001
Remarried	30 (2.7 %)	155 (15.7 %)		
Annual income per capita in the household (CNY^b^)				
<5,000	236 (20.1 %)	340 (32.9 %)	70.902	<0.001
5,000-	338 (28.7 %)	328 (31.7 %)		
10000-	245 (20.8 %)	170 (16.4 %)		
15000-	358 (30.4 %)	196 (19.0 %)		
Quality of relationship with spouse				
Poor	62 (5.2 %)	42 (3.9 %)	2.410	0.300
Average	566 (47.6 %)	528 (49.3 %)		
Good	562 (47.2 %)	501 (46.8 %)		
Living situation				
Not living with the husband’s family	31 (2.6 %)	115 (10.7 %)	61.804	<0.001
Living with the husband’s family	1160 (97.4 %)	956 (89.3 %)		
Number of children				
0	25 (2.1 %)	37 (3.6 %)		
1	698 (59.7 %)	631 (61.3 %)	5.669	0.059
≥2	446 (38.2 %)	362 (35.1 %)		
Number of offspring with current husband				
0	26 (2.2 %)	56 (5.5 %)	34.453	<0.001
1	704 (60.5 %)	684 (66.9 %)		
≧2	434 (37.3 %)	282 (27.6 %)		

^a^ Missing exists if the sum of n is less than N; ^b^ CNY: Chinese Yuan; 6.1 CNY=1 USD

### Social support

The means and standard deviations of social support score received by migrant and non-migrant women are detailed and compared in Table 2. In most aspects immigrant group recorded a lower score, except that the dimension of utilization of support the difference was not statistically significant.

**Table 2:** Comparison of levels of social support between migrant women (*N* = 1,195) and non- migrant women (*N* = 1.076) from rural area of Shandong Province, China

	Migrant women(x ± s)	Local women(x ± s)	*t*	*P*
Subjective support	25.00 ± 4.54	26.55 ± 4.12	-8.453	<0.001
How many friends do you have who can provide help and support to you?	2.74 ± 0.86	2.88 ± 0.86	-4.006	<0.001
How is your relationship with your neighbors?	3.16 ± 0.94	3.34 ± 0.90	-4.669	<0.001
How is your relationship with your colleagues?	3.13 ± 0.91	3.33 ± 0.88	-5.575	<0.001
How is the support you receive from your family members?	15.98 ± 3.58	16.99 ± 3.10	-7.148	<0.001
Objective support	7.50 ± 2.27	8.05 ± 2.64	-5.345	<0.001
What was your living condition in the past year?	3.76 ± 0.73	3.94 ± 0.36	-7.618	<0.001
What have been your sources of economic and practical support in case of emergency?	1.77 ± 1.03	1.97 ± 1.31	-4.222	<0.001
What have been your sources of care when you were in trouble?	1.97 ± 1.14	2.13 ± 1.40	-2.917	0.004
Utilization of support	8.11 ± 1.80	8.22 ± 1.56	-1.457	0.145
What is your way of confiding when you are in trouble?	3.42 ± 0.79	3.52 ± 0.72	-3.108	0.002
How do you seek for help when you are in trouble?	2.86 ± 1.01	2.99 ± 0.99	-3.146	0.002
To what extent are you involved in group (e.g. political organizations, religious organizations, labor unions and student unions) activities?	1.84 ± 1.04	1.71 ± 0.76	3.322	0.001
Total points of social support	40.61 ± 6.19	42.81 ± 6.29	-8.383	<0.001

### Factors associated with social support, and the relation of AIDS and STDs infection to social support among migrant women

#### One-way ANOVA

Corrected means of social support scores were first tested by one-way analysis of variance (One-Way ANOVA) (Table 3). Family average income was found to correlate with every score dimension, namely subjective support (*P* < 0.001), objective support (*P* = 0.001), utilization of support (*P* < 0.001) and total score (*P* < 0.001). Several factors were found to possibly influence one or all dimensions of social support scores received, including education status, family average income, years living in Shandong Province, job engaged, relationship with current husband, the number of offspring with current husband and reason for marrying in Shandong.

**Table 3:** Possible influence factors for social support score- one-way ANOVA

	Subjective support			Objective support			Utilization of support			Total score		
	Mean ± SD	*F/t*	*P*	Mean ± SD	*F/t*	*P*	Mean ± SD	*F/t*	*P*	Mean ± SD	*F/t*	*P*
Education level												
Illiterate^a^	24.83 ± 5.08	3.216	0.041	7.16 ± 2.15	4.318	0.014	7.70 ± 1.63	19.830	<0.001	36.69 ± 6.29	2.957	0.052
Elementary school	25.38 ± 4.59			7.48 ± 2.30			7.97 ± 1.83			40.82 ± 6.48^b^		
Junior middle school and above	24.60 ± 4.06			7.74 ± 2.26^c^			8.57 ± 1.76^c^			40.91 ± 5.50^b^		
Annual income per capita in the household (CNY^d^)												
<5000^a^	24.25 ± 5.27	9.254	<0.001	7.21 ± 2.39	6.324	<0.001	7.49 ± 1.69	38.806	<0.001	38.95 ± 7.03	14.046	<0.001
5000-	25.93 ± 4.62^c^			7.74 ± 2.15^c^			7.98 ± 1.69^c^			41.65 ± 6.24^c^		
10000-	25.44 ± 3.59^c^			8.04 ± 2.03^c^			8.34 ± 1.76^c^			41.81 ± 4.94^c^		
15000-	24.42 ± 3.69			7.61 ± 2.08^b^			9.10 ± 1.67^c^			41.13 ± 5.14^c^		
Years in Shandong												
<5^a^	24.83 ± 3.79	0.973	0.405	7.40 ± 2.17	0.356	0.785	8.47 ± 1.80	4.695	0.003	40.69 ± 5.22	0.079	0.971
5-	25.05 ± 4.21			7.55 ± 2.20			8.16 ± 1.80			40.76 ± 5.68		
10-	24.90 ± 4.84			7.61 ± 2.34			8.06 ± 1.78^b^			40.57 ± 6.64		
15-	25.44 ± 4.94			7.50 ± 2.34			8.13 ± 1.80^c^			40.72 ± 6.74		
Job engaged												
Farming	25.24 ± 4.57	4.893	<0.001	7.54 ± 2.28	0.240	0.811	8.01 ± 1.78	-5.864	<0.001	40.80 ± 6.23	1.516	0.131
Others	23.55 ± 3.60			7.49 ± 2.29			8.97 ± 1.73			40.01 ± 5.49		
Quality of relationship with spouse												
Poor^a^	21.50 ± 5.61	29.642	<0.001	5.31 ± 2.44	21.837	<0.001	7.81 ± 2.06	8.253	<0.001	34.62 ± 7.35	26.761	<0.001
Average	24.39 ± 4.33^c^			7.56 ± 2.10^c^			8.34 ± 1.81			40.28 ± 5.97^c^		
Good	25.95 ± 4.44^c^			7.65 ± 2.31^c^			7.90 ± 1.74			41.50 ± 6.02^c^		
Number of offspring with husband												
0^a^	22.82 ± 4.37	13.346	<0.001	6.80 ± 2.65	2.864	0.057	8.95 ± 1.79	12.125	<0.001	38.57 ± 5.91	4.777	0.009
1	24.68 ± 4.60^c^			7.49 ± 2.25^b^			8.19 ± 1.82^c^			40.37 ± 6.36^b^		
≥2	25.87 ± 4.29^c^			7.59 ± 2.15^b^			7.76 ± 1.73^c^			41.22 ± 5.82^c^		
Reason of marrying in Shandong												
Good living condition	25.01 ± 4.67	3.739	0.024	7.54 ± 2.14^c^	11.003	<0.001	8.03 ± 1.77	4.499	0.011	40.58 ± 6.06^c^	9.612	<0.001
Devotion to husband	25.47 ± 4.31^c^			7.79 ± 2.38^c^			8.38 ± 1.75^b^			41.65 ± 6.17^c^		
Others ^a^	24.30 ± 4.35			6.80 ± 2.31			8.00 ± 1.90			39.10 ± 6.25		

^a^ control group; ^b^ compared with control group *P*≦0.05

^c^ compared with control group *P*<0.01; ^d^ CNY: Chinese Yuan; 6.1 CNY = 1 USD

Corrected means of social support scores were also tested by one-way analysis of variance (One-Way ANOVA) with their self-reported prevalence of AIDS/HIV and sexually transmitted diseases (STDs) (Table 4). Women diagnosed with STDs received lower utilization of support score (*P* = 0.0029) and lower total score (*P* = 0.0171). Compared with the women who did not know their AIDS infectious status, women knowing they were not infected received higher scores in subjective support (*P* = 0.0304), utilization of support (*P* = 0.032) and total score (*P* = 0.0137).

**Table 4:** Possible relation of AIDS and STDs infection to social support score- one-way ANOVA

	Subjective support			Objective support			Utilization of support			Total score		
	Mean ± SD	*F/t*	*P*	Mean ± SD	*F/t*	*P*	Mean ± SD	*F/t*	*P*	Mean ± SD	*F/t*	*P*
Are you infected with AIDS												
Yes	27.67 ± 3.67	3.504	0.030	6.83 ± 2.79	0.887	0.412	8.83 ± 2.48	3.455	0.032	43.33 ± 5.13	4.308	0.014
No	25.11 ± 4.46^b^			7.55 ± 2.24			8.17 ± 1.80^b^			40.83 ± 6.06^b^		
Don’t know ^a^	24.27 ± 4.91			7.34 ± 2.26			7.80 ± 1.66			39.41 ± 6.80		
Have you been diagnosed STDs												
Yes	23.83 ± 4.70	3.608	0.058	7.37 ± 1.99	0.138	0.710	7.42 ± 1.49	8.899	0.003	38.62 ± 6.25	5.700	0.017
No	25.04 ± 4.52			7.50 ± 2.27			8.16 ± 1.80			40.71 ± 6.19		

^a^ control group

^b^ compared with control group *P*≦0.05

### Multi-way ANOVA

Relevant factors of social support scores selected from One-way ANOVA were then analyzed with multi-way analysis of variance (multi-way ANOVA), the correlations of scores to self-reported AIDS and STDs infection were also included. Those possible relevant factors were examined respectively for independent effect. According to the result obtained, several factors were found to correlate with different dimensions of social support score. (Table 5).

**Table 5:** Factors related to dimensions of social support- multi-way ANOVA

Source	Subjective support					Objective support				
	Type III Sum of Squares	df	Mean Square	*F*	*P*	Type III Sum of Squares	df	Mean Square	*F*	*P*
Corrected Model	2340.803	18	130.045	7.385	<0.001	313.105	18	17.395	3.955	<0.001
Intercept	17203.945	1	17203.945	976.997	<0.001	1408.332	1	1408.332	320.217	<0.001
Education level	27.728	2	13.864	0.787	0.455	12.310	2	6.155	1.399	0.247
Income	479.938	3	159.979	9.085	<0.001	83.290	3	27.763	6.313	<0.001
Job	112.470	1	112.470	6.387	0.012	1.647	1	1.647	0.374	0.541
Quality of relationship with spouse	778.727	2	389.363	22.112	<0.001	128.968	2	64.484	14.662	<0.001
Number of children with husband	264.589	2	132.294	7.513	0.001	3.249	2	1.624	0.369	0.691
Reason of marrying in Shandong	84.064	2	42.032	2.387	0.093	33.980	2	16.990	3.863	0.021
Years living in Shandong	16.437	3	5.479	0.311	0.817	2.370	3	0.790	0.180	0.910
Are you infected with AIDS	183.720	2	91.860	5.217	0.006	1.554	2	0.777	0.177	0.838
Have you been diagnosed STDs	30.309	1	30.309	1.721	0.190	0.005	1	0.005	0.001	0.973
Source	Utility of support					Total score of social support				
	Type III Sum of Squares	df	Mean Square	*F*	*P*	Type III Sum of Squares	df	Mean Square	*F*	*P*
Corrected Model	497.359	18	27.631	10.174	<0.001	4541.345	18	252.297	7.775	<0.001
Intercept	2098.429	1	2098.429	772.68	<0.001	46010.323	1	46010.323	1417.886	<0.001
Education level	28.237	2	14.118	5.199	0.006	72.460	2	36.230	1.116	0.328
Income	136.079	3	45,360	16.702	<0.001	1444.949	3	481.650	14.843	<0.001
Job	12.552	1	12.552	4.622	0.032	69.650	1	69.650	2.146	0.143
Quality of relationship with spouse	44.151	2	22.076	8.129	<0.001	1411.830	2	705.915	21.754	<0.001
Number of children with husband	13.839	2	6.920	2.548	0.079	194.533	2	97.266	2.997	0.050
Reason of marrying in Shandong	21.933	2	10.967	4.038	0.018	365.918	2	182.959	5.638	0.004
Years living in Shandong	2.284	3	0.761	0.280	0.840	19.353	3	6.451	0.199	0.897
Are you infected with AIDS	13.488	2	6.744	2.483	0.084	306.878	2	153.439	4.728	0.009
Have you been diagnosed STDs	33.466	1	33.466	12.323	<0.001	125.854	1	125.854	3.878	0.049

Relationship with spouse was positively correlated with scores of all dimensions (*Ps* < 0.001). Migrant women with better relationship with their husband recorded higher scores on subjective and objective support as well as on total score. Moreover, for women with better spousal relationship (medium and good group), utilization of support tended to descend as relationship condition ascends.

Family average income was also found to correlate with all dimensions of scoring. Higher income indicated higher score on support utilization (*P* < 0.001). Migrant women with annual income per capita less than 5000 CNY (the <5000 group) had the lowest subjective support and the 5000–9999 group had the highest one. For the objective support and the total score, the <5000 group scored the lowest while the 10000–14999 group scored the highest. In addition, a positive correlation existed between number of children with current spouse and subjective support as well as total score of support with *P*-values of 0.001, and 0.050 respectively. With higher education, utilization of support score rose significantly (*P* = 0.006). Besides, migrant women engaged in agricultural activities and services received higher scores in subjective support dimension than women with other jobs, while receiving lower scores in utility of support dimension.

When it came to reason for immigration to Shandong among the migrant women, devotion graded higher in objective and utilization part as well as in total score than those holding living condition reasons or other reasons. However, years of living in Shandong recorded no statistical significance in relation to social support scores in all dimensions.

As for AIDS and STDs infection status, it is found that infection of AIDS was correlated with subjective support (*P* = 0.006) and infection status of STDs with utility of support score (*P* < 0.001), while they both had a correlation with total score of social support with *P*-values of 0.009 and 0.049 respectively.

## Discussion

This paper examined the social support condition among a probability-based sample of rural married migrant women and local non-migrant women in Shandong province, China. Compared to their local non-migrant counterparts, migrant women recorded lower scores of social support in almost all dimensions. This finding corresponds with past studies on migrant groups in China [25, 26]. It also confirms the results of one of the few social support study focused on Chinese migrant women [21].

The finding that migrant women had less social support is understandable. Their migration is one from underdeveloped remote areas even less-developed countries to a coastal developed province, thus correlates with many stressors such as language barriers, cultural conflicts, ethnic discrimination, change in social network and lack of support systems, which have been identified as influencing factors of social support for immigrants. Besides, as a special group in the migrant population, migrant women are separated from family and friends, depend more on their current husband and have few links with the new community [27]. This may intensify their weakness in social support network. Considering that their primary reasons for migration are marriage and pursuit of a better life, this is especially true. Migrant women also face special gender-related socioeconomic and physiological problems. Prenatal and postpartum problems are noted among them [28–30]. Stress factors in life as acculturation and adaption may intensify these problems. Thus, they are more susceptible to physical and mental health problem than local women.

However, there are also reports that migrant populations have a higher support level than local groups, arguing that immigration enables people to obtain better job and better socioeconomic status, thus acting positively on them [31, 32]. However, although migrant women have risen in socioeconomic status in our scenario, this theory does not apply. Compared with the widely-focused-on immigrants group (e.g., immigrant workers and merchants) whose migrating destination are developed districts, women in this study have several characteristics which may explain their worse social support conditions: (1) possible ethnic and language barriers makes them unable to smoothly adapt to new communities or pursue better socioeconomic status; (2) their origin and special way of migration (marriage) makes them susceptible to discrimination and hinders them from merging into new local societies, and additionally the conservative atmosphere in rural areas may intensify this discrimination; (3) low socioeconomic status of their husbands in local communities weakens their relative affection and living content.

This study is also the first to appraise common life factors as relevant factors of social support condition among married migrant women. One of the most important factors identified is relationship with spouse. Better relationship is related with higher scores in all dimensions of support, which agrees with the conclusions that family plays a critical role in the mental health of female participants [33] and that immigrant women rely more on their husband [28]. Better relationship indicates more support from spouse and family, which helps immigrants diminish acculturative stress and perceived discrimination during migration and adaptation [34]. However, this study also found that utilization of support declines on a higher level of subjective support score. This may because women with better relationship with spouse feel more content and positive, thus less motivated to seek and utilize more social support. It is also noted that women who have more children with their present spouses scored higher in subjective support and the total score. As children serve as the bond of family members, they can influence the relationship with spouse greatly as well as play a big role in the mothers’ mental health.

Additionally, higher family average income correlates with higher support scores in a certain range, since higher economic level satisfies the family’s material needs better and can provide the family with better social status. Higher education level is related with better utility of social support, possibly because better education indicates better adaptation and self-adjustment. On reasons of moving to Shandong, women claiming devotion had higher grades in objective and utilization part as well as in total score than those holding other reasons. This may because those claiming devotion as the reason are more willing to adapt to new environment and seek for help when necessary.

Number of years living in Shandong however showed no significant relation with social support scores in any dimensions, although it had been expected that as immigrants live longer in the move-in place, their social support develops because of better adaptation, as well as their mental health status.

It has been reported that married migrant women were more likely to have HIV-related behaviors (i.e., blood transfusion, premarital sex, extramarital sex, sexually transmitted diseases and drug use), and less likely to have an HIV test [35]. We find that higher total social support score is related to better awareness of AIDS/STD infection. This finding coheres with the theories that social support as a critical part in psychological health has positive effects on migrants’ health status as well as enhances their utilization of health care services. Migrant women with better social support may carry out less health risk behaviors such as extramarital sex and drug use. They also make better use of health care resources so that their AIDS and STDs infection status can be monitored and controlled. Moreover, we found that higher subjective support scores indicate better awareness of AIDS infection. Considering the characteristic of subjective support, it may suggest that support from families, friends, neighborhoods and colleagues play an important part in self-monitoring of AIDS. Additionally, higher scores in utility of support correlate with less possibility of being diagnosed with STDs, suggesting that individual utilization of available social support facilitates the controlling and prevention of STDs infection.

There are certain limitations in this study. First, because we only recruited migrant women who attended medical check-ups, our analysis is susceptible to bias considering that those who did not attend medical care may be worse off in terms of social support and related outcomes. Second, due to the cross-sectional nature of the study, no causality can be determined; and as such, the findings of this study should be applied with caution. Also, it is important to identify possible influencing factors other than those investigated in this study, which may impact social support and social network as well as migrant women’s mental health. Future studies can include factors such as personal willingness of adaptation, interpersonal relationship, and perceived discrimination and their association with social support level. This can help further increase the understanding of the effects of the socio-psychological factors on social support. Finally, pre-migration factors were not investigated in this study, and these could also have had an influence on immigrants’ adjustments and development of social support condition. Additionally, in the analysis of AIDS infection status, there were only 6 people in migrant women group reported knowing themselves to be infected, which may impact the validity of analysis. Despite these limitations, we believe our study also has several strengths. This study is a pioneering analysis of the marriage-based migrant women who plays a critical role in HIV/AIDS prevalence in their ingoing ground. We picked up representative cities to sketch the social support condition and minimized the cost of the study. Additionally, we confirmed the relation of HIV/AIDS infection to social support condition in this group.

## Conclusions

This study outlines the social support condition as pioneering analysis of the special group of married migrant women who play an important role in HIV/AIDS prevalence in Shandong province, China. Several factors, namely education level, family average income, occupation, relationship with spouse, the number of children with husband, claimed reason of marrying in Shandong, were identified to influence various dimensions of social support. This study also confirmed that higher level of social support is related with better awareness of AIDS and STDs infection among this group. This permits better interference of health professionals and administrators, helping Chinese migrant women improve their social support and health status, thus benefiting the control and prevention of HIV/AIDS. Further research should explore practical ways to provide social and mental support to this special group.

## Abbreviations

AIDS: Acquired Immune Deficiency Syndrome
HIV: Human Immunodeficiency Virus
STD: Sexually Transmitted Disease
ANOVA: Analysis of Variance
